# Diversity of *Bacillus thuringiensis* Strains From Qatar as Shown by Crystal Morphology, δ-Endotoxins and *Cry* Gene Content

**DOI:** 10.3389/fmicb.2018.00708

**Published:** 2018-04-11

**Authors:** Kavita Nair, Roda Al-Thani, Dhabia Al-Thani, Fatima Al-Yafei, Talaat Ahmed, Samir Jaoua

**Affiliations:** Department of Biological and Environmental Sciences, College of Arts and Sciences, Qatar University, Doha, Qatar

**Keywords:** *Bacillus thuringiensis*, crystals, δ-endotoxins profiling, *cry* genes, plasmids

## Abstract

*Bacillus thuringiensis* (Bt) based insecticidal formulations have been recognized as one of the most successful, environmentally safe and sustainable method of controlling insect pests. Research teams worldwide are in search of Bt diversity giving more choices of bio-insecticides and alternatives to address insect resistance. In fact, there are many unexplored ecologies that could harbor novel Bt strains. This study is the first initiative to explore Bt strain diversity in Qatar. A collection of 700 Bt isolates was constructed. Scanning electron microscopy of Bt crystals showed different crystal forms, with a high abundance of spherical crystals compared to the bipyramidal ones. Among the spherical crystals, four different morphologies were observed. The δ-endotoxin content of parasporal crystals from each Bt isolate revealed that there are 16 different protein profiles among the isolates of the collection. On the other hand, plasmid pattern analysis showed seven different plasmid profiles. Their insecticidal activity was predicted by exploring the δ-endotoxin coding genes and conducting qualitative insecticidal bioassays. 19 smooth spherical crystal producing isolates have been identified that could be possible candidates for endotoxin production targeting Dipteran insects. Another group of 259 isolates producing bipyramidal and cuboidal crystals could target Lepidopteran and Coleopteran insects. The remaining 422 isolates have novel profiles. In conclusion, Qatari soil ecology provides a good collection and diversity of Bt isolates. In addition to strains harboring genes encoding common endotoxins, the majority are different and very promising for the search of novel insecticidal endotoxins.

## Introduction

*Bacillus thuringiensis* (Bt) is an entomopathogenic, rod shaped, Gram positive, spore-forming and aerobic bacterium found usually in soil, grain dusts, dead insects and water (Lambert and Peferoen, 1992). During the sporulation stage, they produce parasporal insecticidal protein crystals or δ-endotoxins (Jouzani et al., 2008a,b). Bt is considered the most successful bioinsecticidal alternative available to man today, owing to its toxicity toward a broad range of insect pests such as Dipteran, Lepidopteran and Coleopteran (Federici et al., 2006; Lacey et al., 2015). Based on their serotype and phylogenetic features, Bt is classified into subspecies; which are further classified into serotypes and strains (Seifinejad et al., 2008). Some of the well-studied subspecies include Bt *israelensis* and Bt *kurstaki* that are currently being used as a source for endotoxin protein production at a commercial level (Dambach et al., 2014; Elleuch et al., 2015; Jeong et al., 2017; Zhang et al., 2017). Bt *israelensis* (Bti) produces a combination of endotoxins (Cry) and hemolytic proteins (Cyt) during sporulation stage and crystallize them in a spherical form. On the other hand, Bt *kurstaki* (Btk) produces a combination of other endotoxins (Cry) and crystallize them into bipyramidal form and cuboidal form (Adang et al., 2014). These endotoxins are target specific, for example, the spherical crystals of Bti are toxic to Dipteran insects while the bipyramidal and cuboidal crystals of Btk are toxic to Lepidopteran and Coleopteran insects (Jain et al., 2017). The parasporal crystal form is an indication of its Cry proteins content and is hence used as the first criterion of classification of Bt isolates (López-Meza and Ibarra, 1996; Ben-Dov et al., 1997; Mahalakshmi et al., 2012). As per the database of known endotoxins maintained by Crickmore et al. (2016), there are currently 74 known families of *cry* genes having 770 different *cry* genes and three *cyt* families having 38 *cyt* genes (http://www.lifesci.sussex.ac.uk/home/Neil_Crickmore/Bt/ accessed on 8th October 2017). Apart from Cry and Cyt proteins, Bt also produces other insecticidal proteins called Vip (vegetative insecticidal proteins); which as the name suggests, are expressed during the vegetative stage (Abdelkefi-Mesrati et al., 2011; Abdelmalek et al., 2016) and other useful proteins like Bacteriocins (Jung et al., 2008; Kamouneh et al., 2011). The understanding of the Bt isolates with specific insecticidal genes, becomes even more complex, when one considers that these genes are mostly expressed on plasmids that can be transferred among each other; completely or partially (Rolle et al., 2005). Hence, every ecology might have Bt isolates with unique combinations of insecticidal genes. Research teams worldwide are constantly screening different ecologies to find such novel Bt isolates (Campanini et al., 2012; Soares-da-Silva et al., 2015; El-Kersh et al., 2016).

Knowing that Bt screening programs have never been conducted before in Qatar, this is the first study in the country where 700 Bt isolates were collected from Qatari soil in order to study the diversity and characteristics of Qatari Bt strains. For such big collections of isolates the first criterion of categorization is the crystal shape of the isolates. Ultimately our aim was to study the endotoxins produced by the isolates.

Techniques commonly used, to group and choose a true representative, include plasmid pattern comparisons, PCR (polymerase chain reactions) amplifications of known insecticidal genes, protein analysis, Pulsed-Field Gel Electrophoresis (PFGE), and Ribotyping. (Saadaoui et al., 2010; Sellami et al., 2013; Elleuch et al., 2015). Recent advances in the technologies have seen the use of next generation sequencing and “omics” studies like Genomics, Transcriptomics and Proteomics. (Dong et al., 2016). Although all the techniques have their advantages and limitations, there is always a need to use the right order of these techniques in order to avoid missing out on unique isolates; especially when it comes to big collections of isolates.

## Materials and methods

### Collection, isolation, and preservation of qatari Bt samples

Seven hundred Bt isolates were collected from soil samples in Qatar. Spore-forming isolates were obtained using the acetate selection method from Travers et al. (1987) with slight modifications. One gram of each soil sample was suspended in 10 ml of Luria Bertini (LB) broth, buffered with 250 mM Sodium Acetate (pH 6.8). The mixture was then incubated in a shaker incubator at 30°C for 4 h. After incubation, the samples were heated at 80°C for 15 min. From each of these samples, 100 μl was spread on T3 agar plates and incubated for 72 h at 30°C. Each pure isolate was subjected to microscopic observation to confirm the presence of spores and crystals. Each parasporal crystal forming Bt isolate was named QBT followed by its serial number. Bt isolates were then grown on T3 sporulation media (Travers et al., 1987) for 96 h and the spores-crystal mixture was stored in 30% glycerol at −80°C.

### Identification of crystal morphology

Each sample was grown on T3 agar plates and incubated at 30°C for 96 h. After sporulation, the spore crystal mixture was checked under light microscope to identify the shape of the crystals and group the collection based on their crystal shapes: Bipyramidal, Cuboidal and Spherical. The crystals were then studied in detail under FEI Nova NanoSem 450 Scanning Electron Microscope (SEM), USA to identify intrinsic details and differences between crystals of different isolates. *Bacillus thuringiensis israelensis* (Bti) and *Bacillus thuringiensis kurstaki* (Btk) were used as reference strains.

### Ribotyping based on 16s rRNA gene

The plasmid DNA of of Bt isolates representing the 4 classes forming different spherical crystals was used as template for amplification of 16s rRNA gene using the primer sets Rib73 (5′-AGAGTTTGATCCTGGCTCAG-3′) and Rib74 (5′-AGGAGGTGATCCAGCCGCA-3′). The amplification was carried out using polymerase chain reaction (PCR) in Applied Biosystems 96 wells Veriti Thermal Cycler from Thermo Fisher, USA. The amplified products were run on a 1.2% agarose gel. Expected band (1.5 kb) was gel purified using QIAquick gel extraction kit from Qiagen by following the instruction manual. The purified product was sequenced by Sangers sequencing using 3500 Series Genetic Analyzer by Thermo Fisher Scientific, USA. The 16s ribosomal DNA sequences were submitted to NCBI (National Center for Biotechnology Information) database. The sequences obtained were compared to published sequences on NCBI database.

### Purification of crystal proteins

After complete sporulation, T3 culture with spore crystal mixture of each isolate was centrifuged and the pellet was washed thrice with 1 N NaCl. The pellet was then washed 3x with distilled water. The spore-crystal pellet was re-suspended in 50 mM NaOH and incubated at room temperature for 1 h to solubilize the crystal proteins.

### Studying protein patterns by SDS-PAGE

The purified crystal proteins were combined with 2x boiling buffer (containing 0.1% ß-mercaptoethanol, 1% SDS, 0.025% Bromophenol blue and 10% glycerol) in the ratio 2:1, respectively. The samples were then boiled for 5 min along with a GelPilot broad range protein marker. Samples were loaded on a SDS-PAGE gel with a 10% separating gel and 3% stacking gel. The electrophoresis was run at 100 V for 2 h. The gels were stained with a staining solution containing 0.025% Coomassie Brilliant Blue R250. Destaining was performed overnight with a solution containing Ethanol, Glacial Acetic acid and Water in the ratio 5:7:88, respectively.

### Isolation of plasmid DNA and plasmid profiling

Total plasmid DNA was isolated by alkaline lysis method combined with lysozyme treatment and purified using alcohol precipitation as per Sambrook et al. (1989) with slight modifications. The plasmid profiles were obtained by running on 1% agarose gels with 0.5 μg/ml Ethidium bromide. The gels were loaded with 40 μl of each sample, and run at 10 V overnight in 1% TAE tank buffer. The plasmid patterns of the Bti and Btk were used as references.

### Investigation of hemolytic activity

Blood Nutrient agar plates were prepared by adding 5 ml fresh sheep's blood to the 100 ml autoclaved Nutrient agar medium. The plates were divided into grids and each isolate was inoculated onto the Blood agar plates using sterile toothpicks. The plates were then incubated at 30°C overnight. The zone of clearance around the colony of each isolate were checked and the results were interpreted accordingly.

### PCR amplifications of insecticidal genes

Plasmid DNA was used as template for the PCR amplification of genes encoding Cry and Cyt proteins that are insecticidal to Dipteran, Lepidopteran and Coleopteran insects (Table 1). The PCR amplifications were carried out as per Jaoua et al. (1996) using Applied Biosystems 96 wells Veriti Thermal Cycler from Thermo Fisher. Two groups of primers were used for amplifications. First group consisted of Dipteran specific genes including *cry4A/4B, cry11, cry10, cyt1A, cyt1C, cyt2A, p19*, and *p20. Second* group consisted of Lepidopteran and Coleopteran specific genes including *cry1A, cry1IA, cry1B, cry1D, vip3a*, and *cry2*.

**Table 1:** Primers used in this study for the exploration of genes encoding endotoxin, accessory proteins and Cyt.

**Gene**	**Primers**	**Sequences**	**Reference**
*cry1A*	Lep1A	5′ CCGGTGCTGGATTTGTGTTA 3′	Carozzi et al., 1991
	Lep1B	5′ AATCCCGTATTGTACCAGCG 3′	
*cry1B*	Cry1B1	5′ CTTCATCACGATGGAGTAA 3′	Cerron et al., 1994
	Cry1B2	5′ CATAATTTGGTCGTTCTGTT 3′	
*cry1D*	Cry1D1	5′ CTGCAGCAAGCTATCCAA 3′	Cerron et al., 1994
	Cry1D2	5′ ATTTGAATTGTCAAGGCCTG 3′	
*cry1IA*	Cry5A	5′ ATGAAACTAAAGAATCAAGA 3′	Masson et al., 1998
	Cry5B	5′ ACCTGTGCTATACCATTTCA 3′	
*cry2*	Cry2-1	5′ GTTATTCTTAATGCAGATGAATGGG 3′	Ben-Dov et al., 1997
	Cry2-2	5′ CGGATAAAATAATCTGGGAAATAG 3′	
*vip3a*	Vip1	5′ ATGAACAAGAATAATACTA 3′	Abdelkefi-Mesrati et al., 2005
	Vip3	5′ TTACTTAATAGAGACATCGT 3′	
*cry4A/4B*	Dip2A	5′ GGTGCTTCCTATTCTTTGGC 3′	Carozzi et al., 1991
	Dip1B	5′ ATGGCTTGTTTCGCTACATC 3′	
*cry10*	Cry10-1	5′ ATATGAAATATTCAATGCTC 3′	Porcar et al., 1999
	Cry10-2	5′ ATAAATTCAAGTGCCAAGTA 3′	
*cry11*	Cry11-1	5′ TTAGAAGATACGCCAGATCAAGC 3′	Bravo et al., 1998
	Cry11-2	5′ CATTTGTACTTGAAGTTGTAATCCC 3′	
*cyt1A*	Cyt1A1	5′ GTTGTAAGCTTATGGAAAAT 3′	Zghal et al., 2008
	Cyt1A2	5′ TTAGAAGCTTCCATTAATA 3′	
*cyt1C*	Cyt1C1	5′ CAAAATCTACGGGAGCAAGG 3′	Designed for this study
	Cyt1C2	5′ GGAAGGATCCCTTTGACTTTT 3′	
*cyt2A*	Cyt2A1	5′ AATACATTTCAAGGAGCTA 3′	Guerchicoff et al., 1997
	Cyt2A2	5′ TTTCATTTTAACTTCATATC 3′	
*p19*	p19-1	5′ GCAGGAGGAACATCACCATT 3′	Designed for this study
	p19-2	5′ GGATTTGCTGAGCAGGTCAT 3′	
*p20*	p20-1	5′ TGACGAGGAAACAGAGTATACGA 3′	Designed for this study
	p20-2	5′ TGAAAGGTTAAACGTTCCGATT 3′	

### Protein preparation for insecticidal bioassay

A single colony from 24 h incubated LB agar plates was re-suspended in 50 ml LB broth in 250 ml flask and incubated at 30°C in a shaker incubator for 24 h. The O.D of the culture was checked after 24 h and 50 ml T3 broth was inoculated with this pre-culture such that the starting O.D of all the isolates were 0.1. The T3 broth cultures were incubated at 30°C in shaker incubator for 96 h, ensuring complete sporulation. The broth was centrifuged at 10000 rpm for 10 min and the pellet was re-suspended in 10 ml distilled water. The spore crystal mixture was then diluted 5 times and was used as testing solution for the bioassay.

### Qualitative bioassay of insecticidal activity

Third and fourth instar larvae of *Culex pipiens complex* were used for bioassay. For each test, five larvae were transferred to the test solution. And incubated overnight at room temperature. All the 19 Bti like isolates and the representatives of other classes with spherical crystals were tested for insecticidal activity. The number of larvae that survived was calculated for each Bt isolate δ-endotoxin sample.

## Results

### Characterization of Bt isolates based on crystal morphology and phylogeny

**Figure 1:**
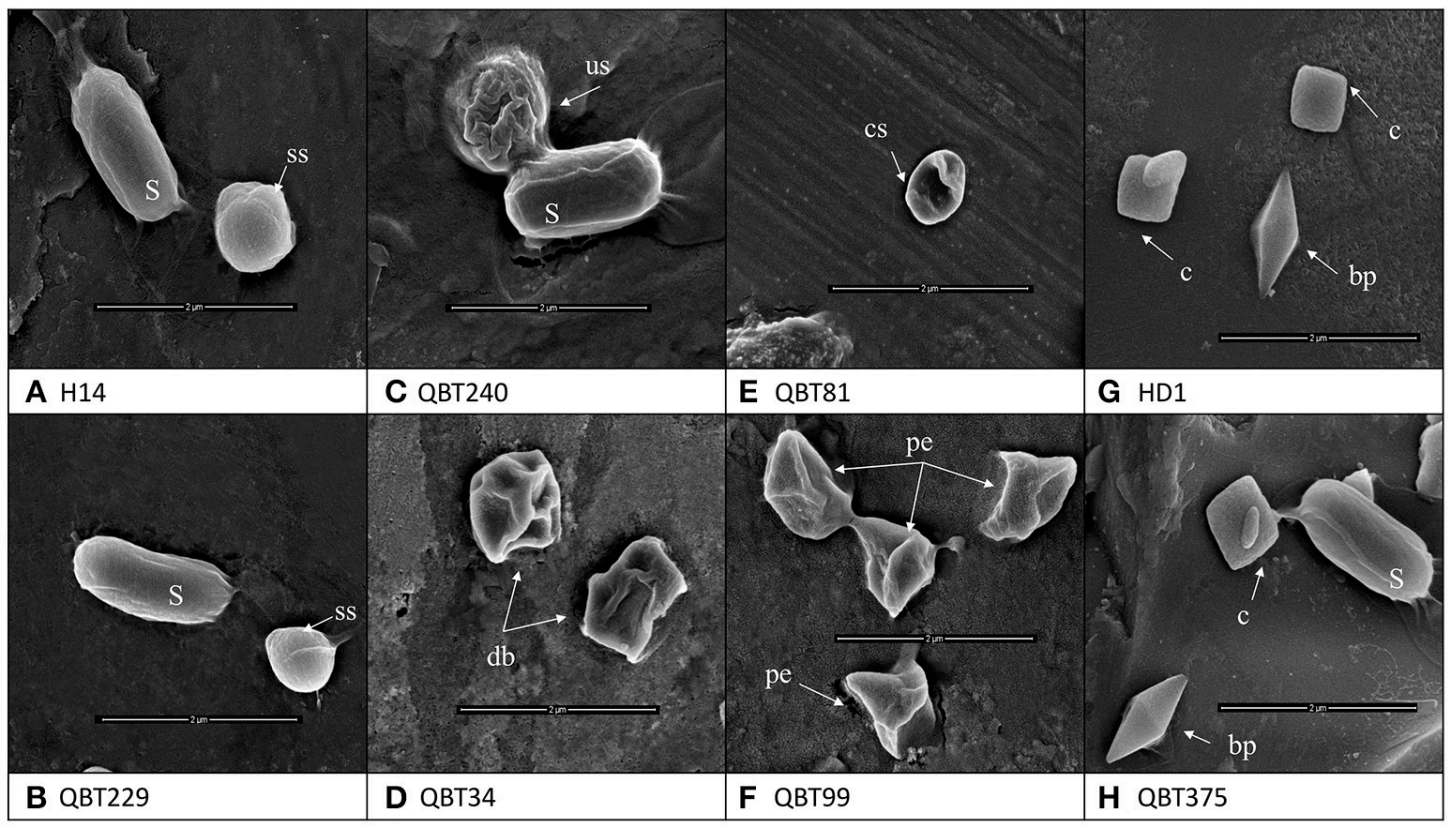
Scanning electron microscopy images of the different types of crystal morphologies and the spores (S) produced by Bt strains of the collection. **(A)** Reference strain *Bacillus thuringiensis israelensis* H14 with smooth spherical [ss] crystal **(B)** Qatari Bti like isolate QBT229 with smooth spherical [ss] crystal **(C)** Spherical crystal with undulated surface [us] QBT240 **(D)** Spherical but deflated balloon [db] shape QBT34 **(E)** Spherical crystals with concave surface [cs] QBT81 **(F)** spherical crystals with pointy edges [pe] QBT99 **(G)** reference strain *Bacillus thuringiensis kurstaki* HD1 with bipyramidal [bp] and cuboidal [c] crystals **(H)** Qatari Btk like isolate QBT375 with bipyramidal [bp] and cuboidal [c] crystals.

The observation of crystal forms of the 700 isolates carried out by light microscope allowed the collection to be classified into two main classes.:441 isolates producing spherical crystals and 259 isolates producing bipyramidal and cuboidal crystals. The crystals were observed by SEM in order to evidence any further differences among the same crystal forms of each class. The results showed that the bipyramidal and cuboidal crystals resembled the standard crystals of the reference Bt *kurstaki* HD1. However, the spherical crystals showed four different shapes when studied at higher magnifications of SEM. In fact, among the collection of 441 spherical crystal producing isolates four groups were identified: smooth spherical (like the reference Bti), spherical with undulated surface, spherical but deflated balloon shape, spherical with one concave side and pointy edged shape (Figure 1).

The plasmid DNA of Bt isolates representing the 4 classes forming different spherical crystals was used for the amplification of the 16s ribosomal DNA using the primers Rib73 and Rib74. The PCR products obtained were purified from the gel using the QIAquick gel extraction kit, following the manual instructions. The purified PCR products were then sequenced by Sangers sequencing. The sequences were submitted to NCBI GeneBank for 16s rRNA and the accession numbers for the same were obtained as MG995012, MG995013, MG995014, MG995015. The *in-silico* analyses of these sequences showed that they had very high (up to 99%) similarity to the published sequences of Bt 16s rRNA genes in the NCBI database. This confirms that the isolates that were identified and characterized in this collection definitely belong to the Bt family.

### δ-endotoxin profiling among Bt isolates

**Figure 2:**
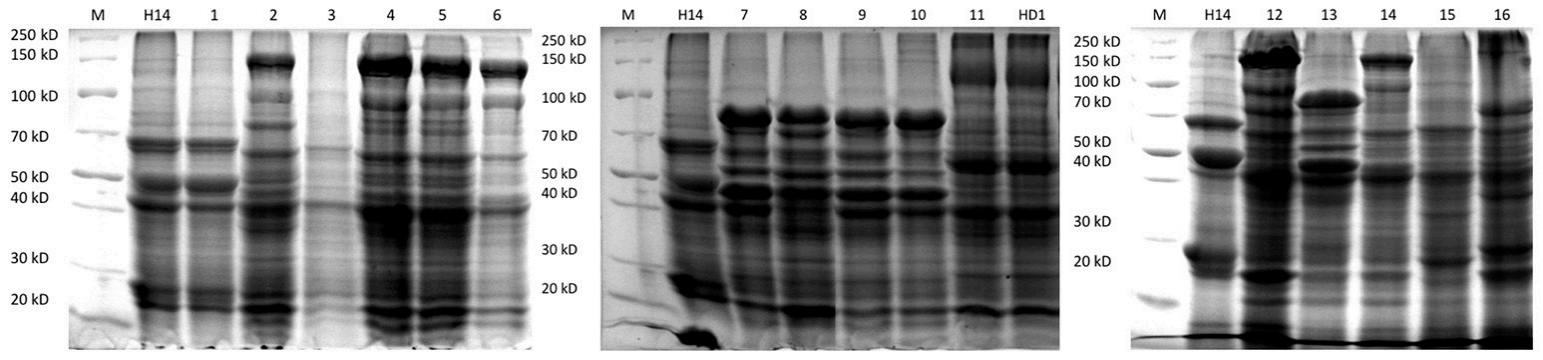
SDS-PAGE gels showing the 16 different protein patterns among the Bt strains collection (1–16). Lanes marked as M is broad range protein marker; H14 is the reference strain *Bacillus thuringiensis israelensis* and HD1 is the reference strain *Bacillus thuringiensis kurstaki*; 1, QBT229; 2, QBT3; 3, QBT6; 4, QBT34; 5, QBT39; 6, QBT212; 7, QBT240; 8, QBT320; 9, QBT418; 10, QBT555; 11, QBT375; 12, QBT41; 13, QBT43; 14, QBT56; 15, QBT81; 16, QBT99.

The protein patterns of the isolates producing bipyramidal and cuboidal crystals were similar to that of the reference subspecies *kurstaki*. They have one protein of about 130 kD, two proteins of about 65 kD and a protein of about 40 kD. However, the spherical crystals harbor different protein patterns when compared to the reference subspecies *israelensis*. In fact, 15 different protein patterns (Figure 2) were evidenced. One protein pattern matched that of the reference Bt *israelensis* H14. These profiles showed mainly the presence of one protein of 130 kD, two proteins of about 65 kD, three proteins of about 45 kD and two proteins of about 27 kD. But 14 other types of protein patterns were observed among the collection. The main proteins as per their sizes are listed in Table 2. Hence, as per the protein patterns, 16 groups (Prot 1 to Prot 16) were evidenced in the collection: 256 Btk like isolates with one protein pattern (Type 11), 19 Bti like isolates with one protein pattern (Type 1) and 14 protein patterns shown by 422 spherical crystals producing isolates (bifurcations shown in Table 2).

**Table 2:** Qatari Bt strains collection summarized into 16 classes with the help of true representatives selected based on crystals morphology, proteomic, and genomic characteristics; QBT229 is the representative of Qatari Bti like isolates, QBT376 is the representative of Qatari Btk like isolates, others represent different spherical crystal morphologies observed with various protein and plasmid pattern.

**Family representative**	**Crystal shape**	**Protein pattern**	**Protein sizes in classes (kD)**	**Plasmid patterns**	**No. of isolates**	**Haemolytic activity**	**Insecticidal activity**	**Bt *cry, cyt* and accessory genes present**
H14	Smooth spherical	–		–		Yes	Yes	*cry4A, cry4B, cry11, cyt1A, cyt2A, cry10, cyt1C, p19, p20*
QBT229		Prot 1	130, 65, 45, 27	Plas 1	19	Yes	Yes	*cry4A, cry4B, cry11, cyt1A, cyt2A, p19, p20*
HD1	Bipyramidal and Cuboidal	–		–		Yes	–	*cry1A, cry1IA, cry1B, cry1D, vip3a, cry2*
QBT376		Prot 11	130, 65, 40	Plas 7	259	Yes	–	*cry1A, cry1IA, cry1B, cry1D, vip3a, cry2*
QBT6	Spherical undulated surface	Prot 3	100, 65, 40	Plas 2	7	Yes	No	–
QBT43		Prot 13	85, 65, 55, 45, 30, 27, 22	Plas 3	33	Slight	No	–
QBT212		Prot 6	130, 90, 60, 40, 22	Plas 4	10	No	No	–
QBT240		Prot 7	100, 80, 60, 45, 40	Plas 4	48	Slight	No	–
QBT320		Prot 8	80, 60, 45, 40, 35	Plas 4	16	Slight	No	–
QBT418		Prot 9	150, 80, 60, 45, 40, 27	Plas 4	9	Slight	No	–
QBT555		Prot 10	180, 150, 80, 60, 45, 40, 27	Plas 4	203	No	No	–
QBT3	Spherical deflated balloon	Prot 2	130, 90, 75, 65, 50, 40, 25	Plas 6	4	No	No	–
QBT34		Prot 4	180, 140, 90, 80, 60, 45, 25	Plas 6	19	No	No	–
QBT39		Prot 5	140, 90, 80, 60, 45, 25	Plas 6	12	No	No	–
QBT41		Prot 12	130, 90, 75, 55, 40, 27, 22	Plas 6	29	No	No	–
QBT56		Prot 14	130, 100, 65, 45, 27, 22	Plas 6	28	No	No	–
QBT81	Spherical concave surface	Prot 15	65, 45, 35, 27	Plas 6	*2*	Yes	No	–
QBT99	Spherical pointy edged	Prot 16	230, 150, 85, 70, 40, 27, 25	Plas 5	*2*	Yes	No	–
				Total	700			

### Distribution of Bt collection based on plasmid patterns

**Figure 3:**
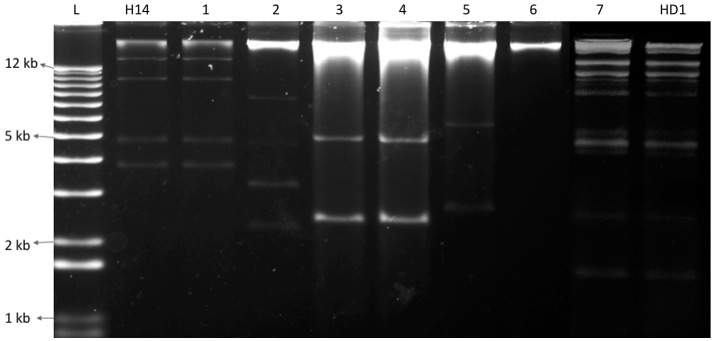
Electrophoresis gel showing seven different plasmid patterns observed among the Bt collection (1–7). L represents a 1 kb plus ladder; H14 is the reference strain Bacillus thuringiensis *israelensis*, HD1 is the reference strain Bacillus thuringiensis *kurstaki*; 1, QBT229; 2, QBT6; 3, QBT43; 4, QBT212; 5, QBT99; 6, QBT3; 7, QBT375.

The investigation of plasmid patterns of the Bt isolates showed that all bipyramidal and cuboidal crystal producing isolates had the same plasmid pattern; similar to that of the reference Bt *kurstaki* HD1. Additionally, the 19 Bti like spherical crystal producing isolates have the same plasmid pattern as that of the Bt *israelensis* H14. However, among the other 422 spherical crystal producing isolates, only 5 types of plasmid patterns were observed. Contrary to the 16 groups of protein patterns in our collection, we have only 7 types (Plas 1 to Plas 7) of plasmid patterns: one Btk like, one Bti like and 5 Non-Bti like (Figure 3). These findings showed that the 700 isolates harbor a total of seven plasmid patterns and 16 different protein patterns (Table 2).

### Hemolytic activity among qatari Bt isolates

**Figure 4:**
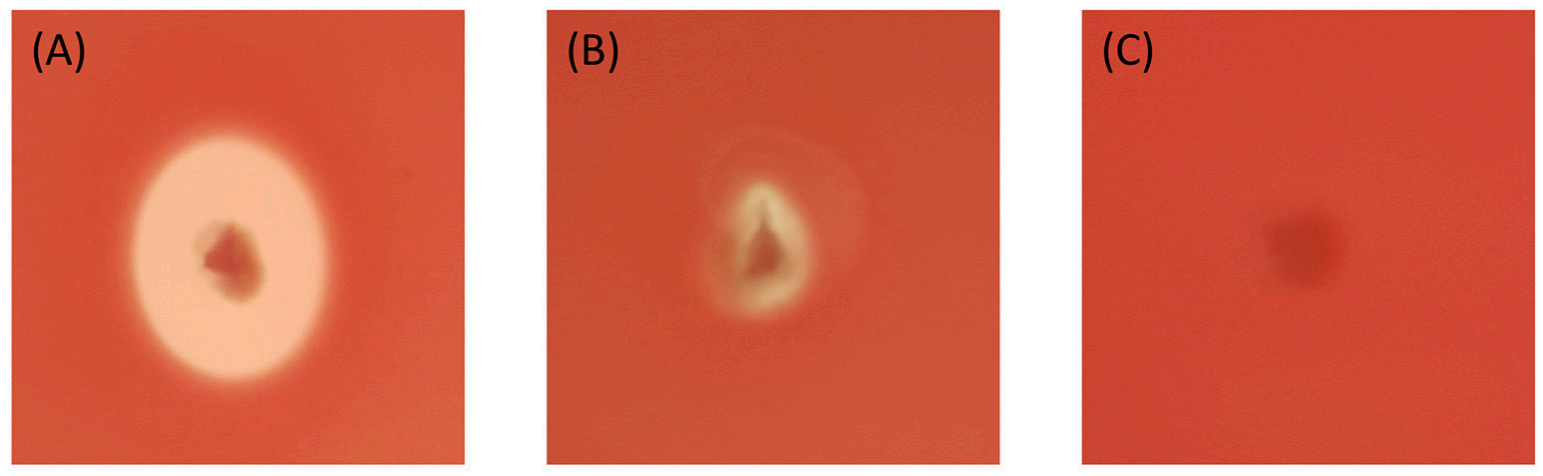
Different types of the hemolytic activities shown by Bt isolates. **(A)** Positive hemolytic activity QBT229 **(B)** Slight hemolytic activity QBT43 **(C)** Negative hemolytic activity QBT3.

The 19 Bti like isolates with smooth spherical crystals, all showed positive hemolysis around their colonies after overnight incubation. The others showed different degrees of hemolysis. Among the crystal forms, the isolates with spherical deflated balloon shaped crystals did not show any hemolytic activity. On the other hand, the isolates with spherical undulated surface crystals showed different hemolytic activities. Some had no activity while some had good or slight activities. The isolates with spherical concave crystals and spherical pointy edged crystals showed good hemolytic activity (Figure 4, Table 2). The isolates with bipyramidal crystals showed hemolytic activity among them like the reference Bt *kurstaki*.

### Insecticidal activity via investigation of δ-endotoxin genes and bioassay

The 19 isolates that resembled the profile of the reference Bt *israelensis* H14 gave the expected PCR product for amplifications with primers designed for exploring genes encoding Dipteran specific endotoxins; except for *cry10* and *cyt1C* genes. Hence, these isolates were tested for their insecticidal activity against *Culex pipiens complex*. The 259 isolates that resembled the profile of the reference Bt *kurstaki* HD1gave all the expected PCR products with primers designed for identifying the presence of genes encoding Lepidopteran and Coleopteran specific endotoxins. The rest of the isolates with spherical crystals did not give expected PCR products with the primers that we tested.

As the 19 Bti like isolates that showed the possible absence of the two important Dipteran specific endotoxin coding genes, the insecticidal bioassay was conducted to check the effect, if any, on the activity. First group consisted of 19 Bti like isolates which were able to kill all the larvae as expected. The second group consisted of 14 representatives that had different protein patterns; they did not show insecticidal activity (Table 2).

## Discussion

Since its first isolation in 1902 by Ishiwata, Bt has been isolated from various ecologies and has been widely studied. In spite of this worldwide exploration, the research on this bacterium is still incomplete (Melo et al., 2016) because of the many ecologies that still remain unexplored and the fact that Bt based insecticides have not been able to completely replace the harmful chemical insecticides in the market. In this study, one such ecology was explored for the first time: Qatari soil. Among the bacterial isolates from soil, 700 isolates were identified as Bt as they all produced parasporal crystals during sporulation. The first level of grouping was done by light microscopy, where 259 isolates were found to produce bipyramidal and cuboidal crystals while the majority (441 isolates) produced spherical crystals. This was contrary to the usual Bt screenings of environmental samples, where the majority produces bipyramidal crystals (Meadows et al., 1992; Bernhard et al., 1997). As Qatar started local agricultural activities very recently, the unavailability of crops and associated insect pests could be a reason for the relatively lower number of Lepidopteran specific Bt strains producing bipyramidal and cuboidal crystals. In this work, Scanning Electron Microscopy was used to not only confirm the crystal shape, but also to further magnify and differentiate between the spherical crystal shapes. Although different types of spherical crystals have been reported before (Noguera and Ibarra, 2010), it has been rarely used as a tool for classifying big Bt collections (Djenane et al., 2017). From SEM images, it was found that among the spherical crystals, there are four kinds of spherical shapes in the collection. This shows the wide diversity among the Qatari Bt strains. The identity of the isolates as Bt strains were confirmed by 16s ribosomal DNA amplification, sequencing and comparing with the published strains from NCBI database. All the isolates belong to the Bt family. When the proteomic content of the parasporal crystals were studied by SDS-PAGE, it was found that isolates with the same crystal shape also had differences among their protein content. In fact, while there are just six types of crystal shapes in the collection, there are 14 types of protein patterns. As the proteins of the parasporal crystals are often encoded by the genes on plasmids, an equal number of plasmid patterns were expected. But, contrary to the published work and theories regarding Bt parasporal crystals (Aptosoglou et al., 1997; Reyes-Ramírez and Ibarra, 2008; Fagundes et al., 2011), only seven plasmid patterns were found among the isolates of the collection. This discrepancy among the protein and plasmid patterns lead to two conclusions. First, the use of plasmid pattern as a tool to identify the diversity among the isolates of an ecology has limitations. In this study, if the plasmid patterns were used as a primary tool of grouping the collection, the actual diversity of the collection wouldn't have been evidenced. Second, as the interest lies in the crystal proteins that have the insecticidal activity, the protein pattern should be ideally used to classify the collection into groups. This will help one understand the real diversity and select true representatives for molecular and genomic studies.

The hemolytic activity of the strains representatives (Bti like and Btk like isolates) was tested and the collection showed varying degrees of hemolytic activities as expected. Among them, the interesting group was the isolates with spherical crystals having undulated surface. The representatives of this group, had varying hemolytic activities. This single group has isolates with good hemolytic activities, slight hemolytic activity and no hemolytic activity. This shows for the first time, the polymorphism among the isolates with same crystal forms.

PCR amplifications were carried out with primers designed to explore *cry* and *cyt* genes, in order to predict their insecticidal activity. Among the Bt collection, 19 isolates resemble *Bt israelensis* H14 in crystal morphology, plasmid pattern and protein pattern. PCR amplifications showed that they carry the Dipteran specific insecticidal protein coding genes like *cry4A/4B, cry11, p19, p20, cyt1A*, and *cyt2*. These 19 isolates among our collection would be the candidates to be tested for their insecticidal activities against Dipteran insects. All these Qatari Bti like isolates failed to give the PCR amplifications for primers designed for *cry10* and *cyt1C* gene. Knowing that these two genes are located next to each other on pBtoxis plasmid of Bti (Berry et al., 2002), it was proposed that there is some kind of structural instability among the Qatari Bti like isolates in this plasmid region. Hence, it was necessary to check the insecticidal activity against Dipteran insects. As shown in Table 2, these Qatari Bti like isolates were able to kill all five larvae of *Culex pipiens*, like reference strain *Bt israelensis* H14. The insecticidal activity still persists among these 19 isolates, in spite of the possible absence of *cry10* and *cyt1C* genes. The exploration of the concerned region of the plasmid will be carried out in further genomic studies to understand the structural instability.

The isolates producing bipyramidal and cuboidal crystals carry all the Lepidopteran and Coleopteran specific insecticidal protein coding genes that were tested by PCR. They harbor *cry1A, cry1IA, cry1B, cry1D, vip3a*, and *cry2*. These 259 isolates producing bipyramidal and cuboidal crystals harboring the gene encoding the essential δ-endotoxins are the candidates for bio-larvicidal production against Lepidopteran and Coleopteran insects. Other 422 isolates gave no amplifications for all the primer sets that were tested so far. This leads to the conclusion that the 422 isolates either have new δ-endotoxin genes and/or different forms of the known endotoxin genes, which could not be detected by PCR. They are very promising in the search of novel endotoxins. These isolates will be studied further on genomic as well as proteomic levels to identify the types of delta-endotin genes harbored by them.

**Figure 5:**
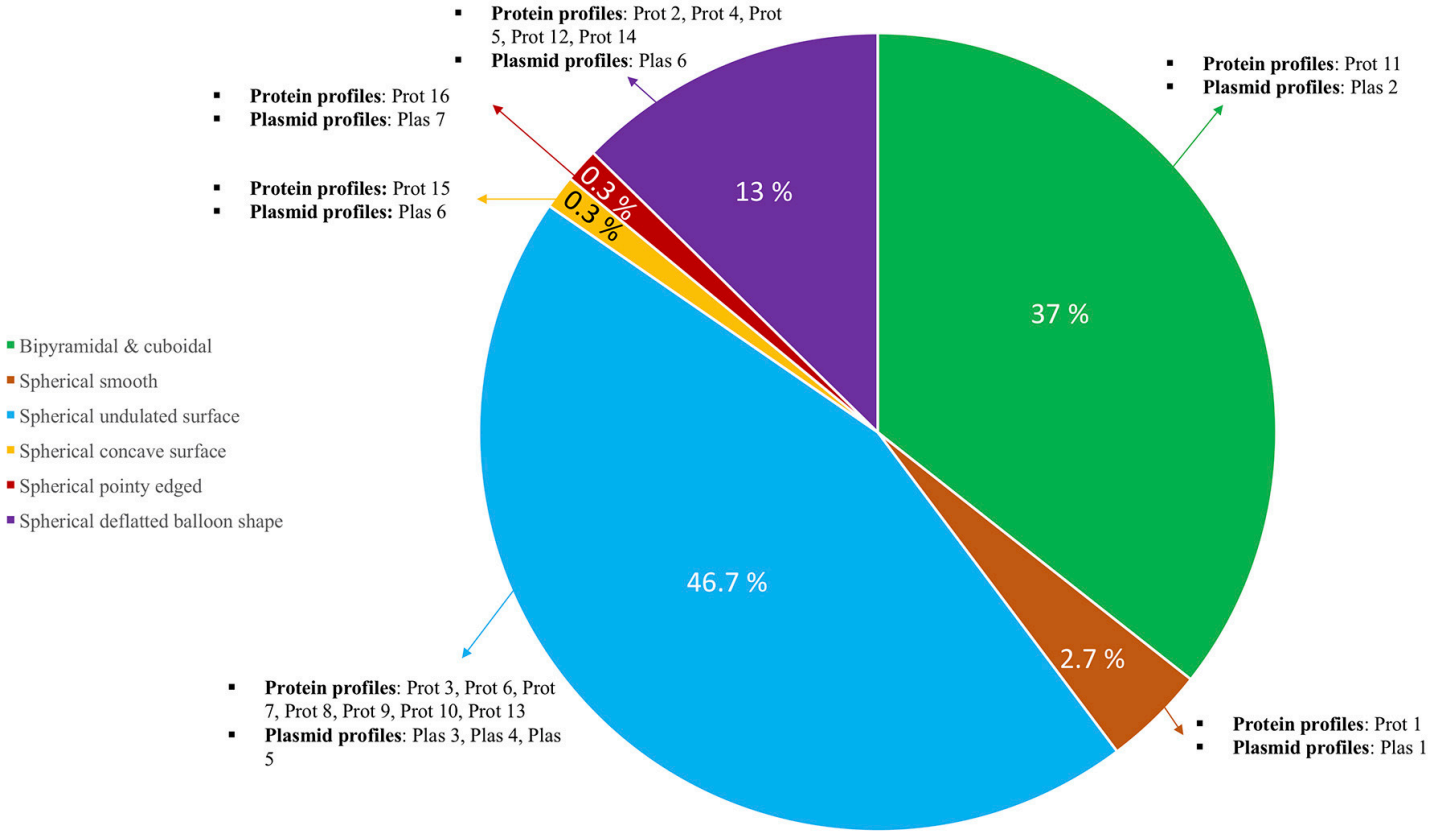
Pie chart showing the diversity among 700 Qatari Bt isolates. Main distribution in the pie is based on crystal forms and each section has been further classified based on the types of protein and plasmid profiles observed among them.

In conclusion, in the first study of Bt screenings in Qatar, the 700 isolates were characterized and grouped into 16 classes (Figure 5, Table 2). Qatar's microbial community offers a good diversity of Bt isolates that could be potential candidates for local production of bio-larvicides against many insect families. The parasporal crystal morphology and protein content of the crystals were given more importance when classifying them. This study shows the advantages of using proteomic techniques over genomic techniques when screening and studying big collections of Bt. We believe that from each group, the representatives that we chose will truly embody its respective group and make it easier to run genomic and proteomic studies accurately with them in the future.

## Author contributions

KN, RA-T, DA-T, FA-Y, TA, and SJ made considerable contribution to the design of the work, procurement of the data and planning the work. KN and SJ made the analysis and interpretation of data, wrote the final version of this manuscript, are accountable for all of the work in ensuring the accuracy and reliability of the work.

### Conflict of interest statement

The authors declare that the research was conducted in the absence of any commercial or financial relationships that could be construed as a potential conflict of interest.

## Abbreviations

Bt: *Bacillus thuringiensis*
Bti: *Bacillus thuringiensis israelensis*
Btk: *Bacillus thuringiensis kurstaki*
PCR: Polymerase chain reactions
PFGE: pulsed field gel electrophoresis
Plas: plasmid pattern
Prot: protein pattern
SDS-PAGE: sodium dodecyl sulfate-polyacrylamide gel electrophoresis.
